# Dr. Nitya Anand: The Visionary Behind Saheli and the Advances in Medicinal Chemistry

**DOI:** 10.7759/cureus.68208

**Published:** 2024-08-30

**Authors:** Anshu Singh, Umesh Kawalkar, Amar Mankar, Manoj S Patil

**Affiliations:** 1 Community Medicine, Government Medical College (GMC) Akola, Akola, IND; 2 Community Medicine, Datta Meghe Institute of Higher Education and Research, Wardha, IND; 3 Research and Development, Jawaharlal Nehru Medical College, Datta Meghe Institute of Higher Education and Research, Wardha, IND

**Keywords:** historical vignette, streptomycin, leprosy treatment, centchroman, family planning method, oral contraceptive pill (ocp), saheli. non-steroidal, padma shree

## Abstract

Dr. Nitya Anand was a leading figure in Indian pharmaceutical research. His career spanned several decades, during which he significantly contributed to advancement in drug development and public health. His innovation of Centchroman (Saheli), the world's first non-steroidal oral contraceptive pill, changes the course of contraception use in India. He had done groundbreaking work in leprosy treatment, synthetic peptides, and antibiotics. With over 400 publications and 130 patents, he supervised over 100 PhD students. His work has been recognized with many prestigious awards, including the Padma Shri and the National Nehru Science Award, which have left a long-lasting impact on medicinal chemistry and public health.

## Introduction and background

**Figure 1:**
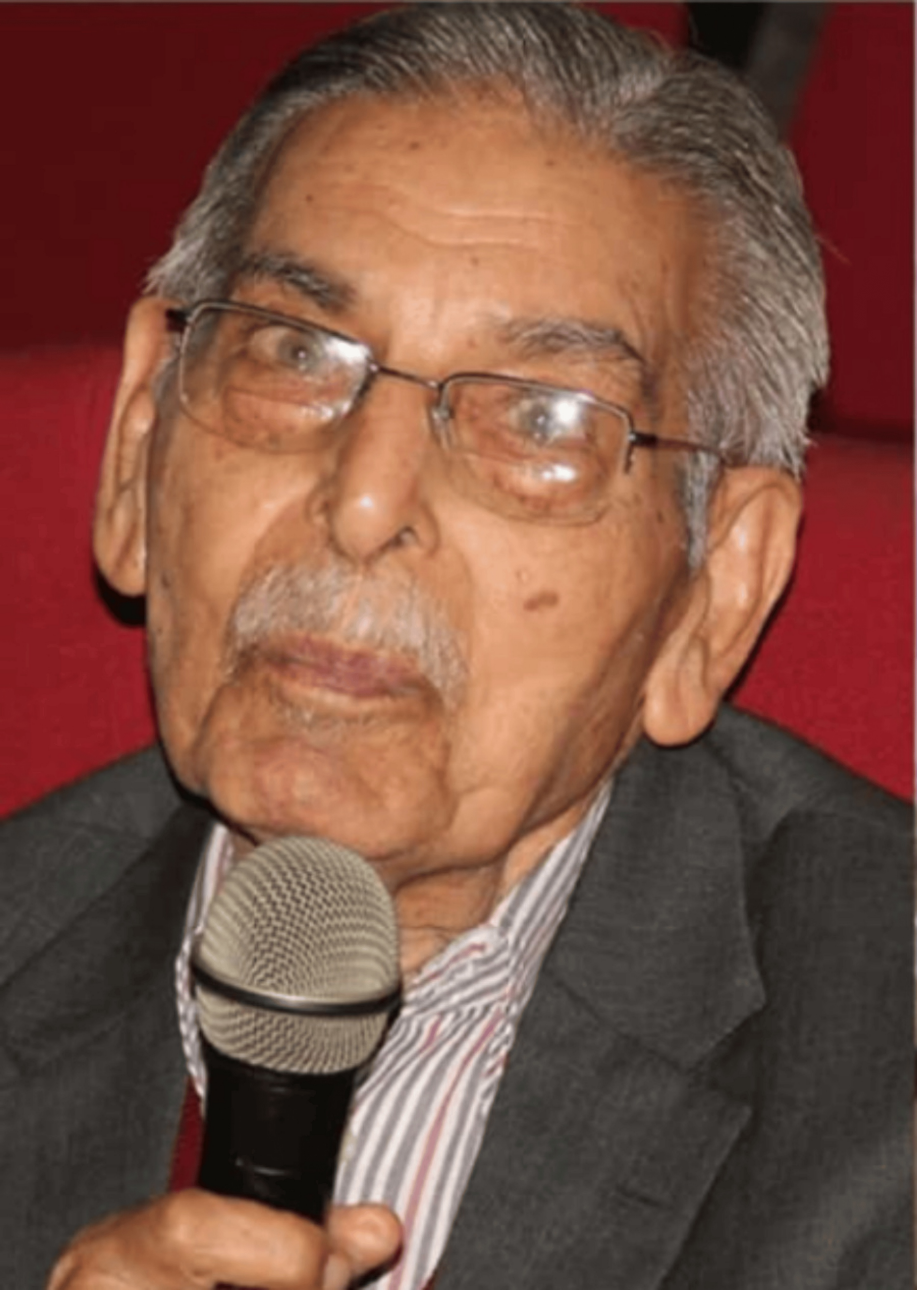
Dr. Nitya Anand (1925-2024) Image courtesy: Wikimedia Commons [1]

Dr. Nitya Anand (Figure 1) [1] is a prominent figure in the history of Indian pharmaceutical research, with his tremendous contribution to the field of medical chemistry and leaving behind his undeniable legacy for future medicinal chemistry. Dr. Anand was born on January 1, 1925, in Lyallpur, West Punjab (now in Pakistan). He served several decades of his long career, contributing to the enhancement of medicinal chemistry and drug development. His work is beyond the labs and has had a tangible effect on pharmaceutical research in India. His contributions to medical chemistry research not only intensified the understanding of drugs but also refined public health by discovering new drugs and molecules [2].

## Review

Early life and education

Dr. Nitya Anand belonged to a family that had education and social work running deep in its tradition. His father was a professor of physics and mathematics, and his mother was the honorary principal at an institution that provided training to widows and marginalized women. His parents were actively involved in social work and the national freedom movement, which embedded the principles of service to humanity in his life [2]. He attended Dhanpatmal Anglo-Sanskrit High School and completed his high school education at Government Inter College in Lyallpur. He completed his B.Sc. from Government College University of Lahore. He went to Delhi for his masters as M.Sc. in Chemistry from St. Stephen's College and followed further research into organic chemistry at the University Department of Chemical Technology, Bombay, where he was awarded his Ph.D. in 1948. He was awarded a second Ph.D. from St. John's College at Cambridge University [3]. He returned to India in late 1950 and worked at Delhi University (DU) in his early career. Then, working for some period, he left the DU and joined the Medicinal Chemistry Division of the newly inaugurated Central Drug Research Institute first as a scientist in 1951. To enhance his drug development and research skills, he joined Harvard Medical School as a postdoctoral scientist in 1958 [2-4].

Contributions of Dr. Nitya Anand

Development of Saheli

Dr. Anand started his career at the Central Drug Research Institute (CDRI) in March 1951, where he worked in different domains as a scientist to the director of ICDR until retiring from his service on December 31, 1984. Under his leadership, CDRI became one of the finest drug discovery and development centers. Centchroman, or "Saheli," was the most famous invention of Dr. Nitya Anand [4-6]. It is the world's very first and only non-steroidal oral contraceptive pill; it acts as a beacon to the contraceptive world in India [3]. Launched by then Prime Minister Rajiv Gandhi in 1986, Saheli was a major and novel innovation in reproductive health options, as it provides an acceptable, safe, and effective contraceptive method. This led to its introduction in the National Family Planning Program of India in 2016, highlighting its crucial importance to date [2].

Research on Leprosy Treatment and Synthetic Peptides

For the treatment of leprosy, Dr. Anand initiated and led the groundbreaking work of designing and synthesizing sulfones and sulfonamides [2]. By adopting a multidisciplinary approach, he effectively utilized his knowledge of key elements in drug design, including membrane transport, the biology of the parasite, and disease pathology. His studies on drug absorption, metabolism, and distribution provided a strong foundation for future advancements in this field [7]. CDRI emerged as the frontrunner in Dr. Anand's leadership in the synthesis of synthetic peptides and a wide range of heterocyclic compounds. The synthesis of potential drugs increasingly became guided by the principles of drug design, taking into account factors such as drug-receptor interactions, metabolism, and pharmacokinetics. His crucial role in the development of muramyl-peptide analogs, nucleosides, and numerous heterocyclic prototypes, including chromenes, diazabicyclo-octanes, and others, cannot be denied [8]. His research on the understanding and development of drugs for central nervous system (CNS) and cardiovascular system (CVS) disorders, parasitic diseases, and fertility regulation has played a major role in today's pharmaceutical world [8-10].

Breakthroughs in Antibiotic Mechanisms and Drug Design

His work in antibiotic mechanisms, particularly streptomycin, is highly valued. His observational studies on streptomycin led to the discovery that streptomycin induces bidirectional leakiness in the membrane of *Escherichia coli*. He conducted this research at Harvard Medical School as a fellow of the Rockefeller Foundation [11]. His research helps establish the strong connection between membrane damage and protein synthesis, enhancing the knowledge of antibiotic mechanisms and their synergistic interactions. Dr. Anand was one of the first to promote and implement the design of prototype molecules with fixed geometry to better understand drug-receptor interactions [12]. His understanding of drug-receptor binding sites and the structure-activity relationship of molecules contributed to the development of several new chemical entities (NCEs), including Centbucridine (a local anesthetic) and Gugulipid (a hypolipidemic agent) [12].

Achievements and Awards

Dr. Anand has published over 400 research papers and held over 130 national and international patents. He had published on various medicinal chemistry and drug design topics, significantly enhancing the scientific community's knowledge base. He has supervised over 100 PhD scholars in his entire working profession, and many of his students have advanced to senior research roles in both academia and the pharmaceutical industry. His guidance played a crucial role in shaping the future of the next generation of medical chemists and research scientists [2]. He was a co-author of Jasjit S. Bindra in the book "Art in Organic Synthesis," which was published in 1969 [3]. His contribution to medical sciences has been recognized with numerous prestigious awards and honors, including the Padma Shri, the Amrut Mody Research Award, the Vishwakarma Medal, and the National Nehru Science Award. He has been honored with the K.G. Nayak Gold Medal, Baroda University; the J.B. Chatterji Gold Medal, Tropical School of Medicine, Calcutta; the Acharya P.C. Ray and Sir J.C. Ghosh Medals, Indian Chemical Society; and the Vigyan Gaurav Award of UPCST. He was a fellow of numerous prestigious academies, including the Indian National Science Academy and the National Academy of Sciences, India [2-4].

## Conclusions

Dr. Nitya Anand's legacy was defined by his innovation, dedication, and excellence in drug research. His exceptional work and leadership at the CDRI of India not only made an impact globally but also elevated the status of the Indian pharmaceutical industry on a global platform. Dr. Anand's commitment to scientific rigor and societal benefit continues to inspire future generations of researchers and scientists. His contributions have left a continuing impact on the field of medicinal chemistry and public health, ensuring his place as an admirable figure in the annals of scientific history.

## References

[1] (2024). Wikimedia Commons. https://commons.wikimedia.org/wiki/File:Nitya_Anand.png.

[2] Saxena AK, Gupta CM (2005). Nitya Anand: a tribute. ARKIVOC.

[3] (2024). Dr Nitya Anand, brain behind India's first oral contraceptive, dies at 99. https://timesofindia.indiatimes.com/city/lucknow/dr-nitya-anand-brain-behind-indias-first-oral-contraceptive-dies-at-99/articleshow/107192140.cms.

[4] Kole P, Ray S, Kamboj VP, Anand N (1975). Studies in antifertility agents. J Med Chem.

[5] Salman Md, Ray S, Agarwal AK (1983). Antifertility agents (XXXVIII). The effect of the side chain and its position on the activity of 3,4-diaryl-chromans. J Med Chem.

[6] Ray S, Grover PK, Kamboj VP (1976). Studies in antifertility agents: part XII - structure activity relationship studies of 3,4-diphenylchromenes and chromans. J Med Chem.

[7] Anand N (1975). Sulfonamides and sulfones. Mechanism of Action of Antimicrobial and Antitumor Agents.

[8] Saxena R, Iyer RN, Anand N, Chatterjee RK, Sen AB (1970). 3-Ethyl-8-methyl-1,3,8-triazabicyclo(4,4,0)decan-2-one: a new antifilarial agent. J Pharm Pharmacol.

[9] Rastogi SN, Anand N, Prasad CR (1972). Agents acting on the central nervous system. XIV. 1-(p-alkanoylphenoxy)-3-(N4-arylpiperazinyl)propan-2-ols. A new class of anti depressants. J Med Chem.

[10] Rastogi SN, Anand N, Gupta PP, Sharma JN (1973). Agents acting on the CNS. Part XIX. (+) 1-(o- and m-alkanoylphenoxy)-3-(N4arylpiperazinyl)propan-2-ols as local anaesthetics, hypotensives and tranquilisers. J Med Chem.

[11] Anand N, Davis BD (1960). Damage by streptomycin to the cell membrane of Escherichia coli. Nature.

[12] Saxena AK, Jain PC, Anand N (1973). Agents acting on the central nervous system-XV, 2-substituted 1,2,3,4,6, 7,12,12a-octahydroprazino(2′,1′:6,1)pyrido(3,4-b)indoles A new class of central nervous system depressants. J Med Chem.

